# fMRI findings in MTBI patients with headaches following rTMS

**DOI:** 10.1038/s41598-021-89118-2

**Published:** 2021-05-05

**Authors:** Michael Vaninetti, Mike Lim, Aladdin Khalaf, Valerie Metzger-Smith, Matthew Flowers, Alphonsa Kunnel, Eric Yang, David Song, Lisa Lin, Alice Tsai, Roland Lee, Shahrokh Golshan, Albert Leung

**Affiliations:** 1 Anesthesiology, UC San Diego School of Medicine, La Jolla, CA 92093 USA; 2 Center for Pain and Headache Research, VA San Diego Healthcare System, San Diego, CA 92161 USA; 3 Anesthesia Pain Service, VA San Diego Healthcare System, San Diego, CA 92161 USA; 4 VA San Diego Healthcare System, San Diego, CA 92161 USA; 5 Physical and Rehab Medicine, VA San Diego Healthcare System, San Diego, CA 92161 USA; 6 Radiology, UC San Diego School of Medicine, La Jolla, CA 92093 USA; 7 VA San Diego Healthcare System, San Diego, CA 92161 USA; 8 Biostatistics Core, VA San Diego Healthcare System, San Diego, CA 92161 USA

**Keywords:** Randomized controlled trials, Medical research, Brain injuries, Headache

## Abstract

Mild Traumatic Brain Injury (MTBI) patients with persistent headaches are known to have diminished supraspinal modulatory connectivity from their prefrontal cortices. Repetitive transcranial magnetic stimulation (rTMS) is able to alleviate MTBI-related headache (MTBI-HA). This functional magnetic resonance imaging (fMRI) study assessed supraspinal correlates associated with the headache analgesic effect of rTMS at left prefrontal cortex (LPFC), hypothesizing real rTMS would significantly increase modulatory functions at LPFC in comparison to sham treatment. Subjects with MTBI-HA were randomized to receive either real or sham rTMS treatments and subjected to pre- and post-treatment resting state and evoked heat-pain fMRI as described in a prior study. Real rTMS consisted of 2000 pulses delivered at 10 Hz and 80% of the resting motor threshold at left dorsolateral prefrontal cortex, whereas sham treatment was delivered with same figure-of-eight coil turned 180 degrees. Follow-up fMRI was performed one-week post-treatment. All fMRI data was processed using BrainVoyager QX Software. 14 subjects receiving real and 12 subjects receiving sham treatments completed the study. The REAL group demonstrated significant (*P* < 0.02) decreases in headache frequency and intensity at one week following treatment. fMRI scans in the REAL group showed increased evoked heat pain activity (*P* < 0.002) and resting functional connectivity (*P* < 0.0001) at the LPFC after rTMS. Neither this significant analgesic effect nor these fMRI findings were seen in the sham group. Sham treatment was, however, associated with a decrease in resting state activity at the LPFC (*P* < 0.0001). This study correlates the demonstrated analgesic effect of rTMS in the treatment of MTBI-HA with enhanced supraspinal functional connectivity in the left prefrontal cortex, which is known to be involved in “top-down” pain inhibition along the descending midbrain-thalamic-cingulate pathway.

**Trial Registration**: This study was registered on September 24, 2013, on ClinicalTrials.gov with the identifier: NCT01948947. https://clinicaltrials.gov/ct2/show/NCT01948947.

## Introduction

Traumatic brain injury (TBI) is a leading cause of morbidity, mortality, and disability worldwide^1,2^. The majority of traumatic brain injuries are classified as mild traumatic brain injuries (MTBI) based on clinical presentation and a variety of classification systems^3^. MTBI can cause cognitive impairment, headache, nausea, and alterations in mood, among other symptoms. Unfortunately, medication management for this condition has shown limited efficacy, while the medications most often used can cause significant physical, psychosomatic, and abusive side effects^4–6^. In addition to pain and suffering on an individual level, the syndrome also carries a large societal impact in the form of loss of days of work, reduced productivity, and loss of employment, especially since it is most prevalent in younger individuals.

Neuropathic pain results from damage, disease, or dysfunction of the somatosensory nervous system. This dysfunction can involve cerebral, spinal, or peripheral pain signaling or modulating pathways, or a combination of these. There is increasing evidence indicating a neuropathic mechanism underlying MTBI-related headache (MTBI-HA)^7–9^. Specifically, our group previously demonstrated diminished supraspinal pain modulation in patients suffering from MTBI-HA^10^. These supraspinal modulatory functional changes are thought to be mediated by several processes, including microscopic diffuse axonal injury limiting communication between cerebral pain modulatory regions, reduced cortical excitability and conductivity in these regions as evidenced by increased resting motor thresholds, and relative hypoperfusion of key cortical relay regions such as the basal ganglia, which is responsible for messaging between pain modulatory cortical areas and the limbic system^11–14^. These phenomena affecting pain modulation, especially with regard to the prefrontal cortices (PFC) with correlated white matter tract deficits, are hypothesized to play an important part in the development of head pain states in this patient population, and further add to the notion that MTBI-HA is a true neuropathic pain state^15^.

Repetitive transcranial magnetic stimulation (rTMS) is being increasingly shown to be effective and is gaining clinical traction as a viable treatment for a variety of neuropathic pain states, one of which is MTBI-HA. In addition to demonstrating reduced supraspinal pain modulation in MTBI-HA patients, our group also previously demonstrated that rTMS can reduce headache symptoms and provide transient mood enhancement in these patients^10,15^. The current study aims to investigate whether these symptomatic improvements following rTMS also correlate with increased, or restored, supraspinal pain modulatory functional connectivities. Such an effect could explain a therapeutic mechanism of rTMS in MTBI-HA. Furthermore, if such changes are found, this study aims to characterize which supraspinal modulatory areas are most impacted by the treatment, advancing our understanding of the mechanisms behind this difficult to treat neuropathic pain condition, and potentially identifying additional cortical regions as treatment targets for rTMS. We hypothesized that real rTMS at PFC would significantly increase functional connectivity at the PFC in comparison to sham treatment. In addition, this increase of activity would correlate with MTBI-HA symptom improvement in response to the real treatment.

## Methods

A single-center, two-arm, interventional randomized clinical trial was conducted with parallel assignment in which subjects were blinded to the treatment group. All assessments and treatments were conducted at a veteran’s hospital and MRI scans were conducted at the hospital affiliated university in California, USA.

### Initial recruiting

Patients who had previously been given a diagnosis of MTBI-HA by a licensed neurologist in a dedicated TBI clinic were subsequently consented, screened, and enrolled by the current study principal investigator (PI) based on the study protocol approved by the Institutional Human Subject Protection Committee. All methods were carried out in accordance with the approved protocol and good clinical practices. Individuals were recruited with the following inclusion criteria: male or female age between 18 to 60 (maximum age reduced from 65 to 60 as compared to original clinical study to minimize age-related fMRI changes); history of MTBI; and the established diagnosis of chronic post-traumatic headache due to mild head injury based on the ICHD-2^16,17^ diagnostic criteria as follows (ICHD-2 was the most recent set of criteria at the time of the study):

A. Headache, no typical characteristics known, fulfilling criteria C and D.
B. Head trauma includes the following:
  1. Either no loss of consciousness or loss of consciousness of <30 minutes in duration
  2. Glasgow Coma Scale (GCS) >13
  3. Symptoms and/or signs diagnostic of concussion as discussed in the below diagnostic criteria of MTBI
C. Headache occurs within 7 days after head trauma.
D. Headache persists for > 3 months after head trauma.

Additional headache inclusion criteria consisted of an average chronic persistent daily (24/7) headache intensity greater than 30 on a 0–100 mechanical visual analog scale (M-VAS) at the screening visit (Visit 1)^18^; and an average intensity of this chronic persistent headache greater than 3/10 on a numerical rating scale (NRS) reported in the headache diary filled out daily by the subjects between Visit 1 and their MRI scans at Visit 2. MTBI diagnosis was based on the published criteria from the 1993 American Congress of Rehabilitation Medicine and recent recommendation from the Department of Defense.

Exclusion criteria included: history of pacemaker implant; pregnancy; ferromagnetic material such as shrapnel, bullet fragments or implanted devices in the body, incompatible with MRI procedures; history of life-threatening diseases, dementia or major psychiatric illnesses; documented diagnosis of Post-traumatic Stress Disorder (PTSD) or Mississippi Scale for PTSD score greater than or equal to 130; documented Major Depression or Hamilton Rating Scale for Depression score greater than or equal to 19; presence of any other chronic neuropathic pain states or neurological diseases such as seizure; involvement of litigation; inability to understand the study instruction and communicate in English; and history of chronic headache diagnoses such as migraine, tension or cluster headaches prior to incidence of MTBI. Subjects who met the above exclusion criteria for depression or PTSD were excluded because of the concern for effects of these conditions on cortical resting state activity^19,20^. Subjects who met the criteria for participation were enrolled by the principal investigator and were randomized into one of two treatment groups, real or sham, by the biostatistician. Sample size was determined based on the number of new potential subjects seen in the TBI clinic at the hospital, the duration and amount of funding, and the number of subjects needed for sufficient statistical power. Specifically for the fMRI subgroup analysis performed for the current study as compared to the broader, prior clinical study, sample size was also determined based on generally accepted and validated sample size minimums for fMRI studies^21^.

**Figure 1:**
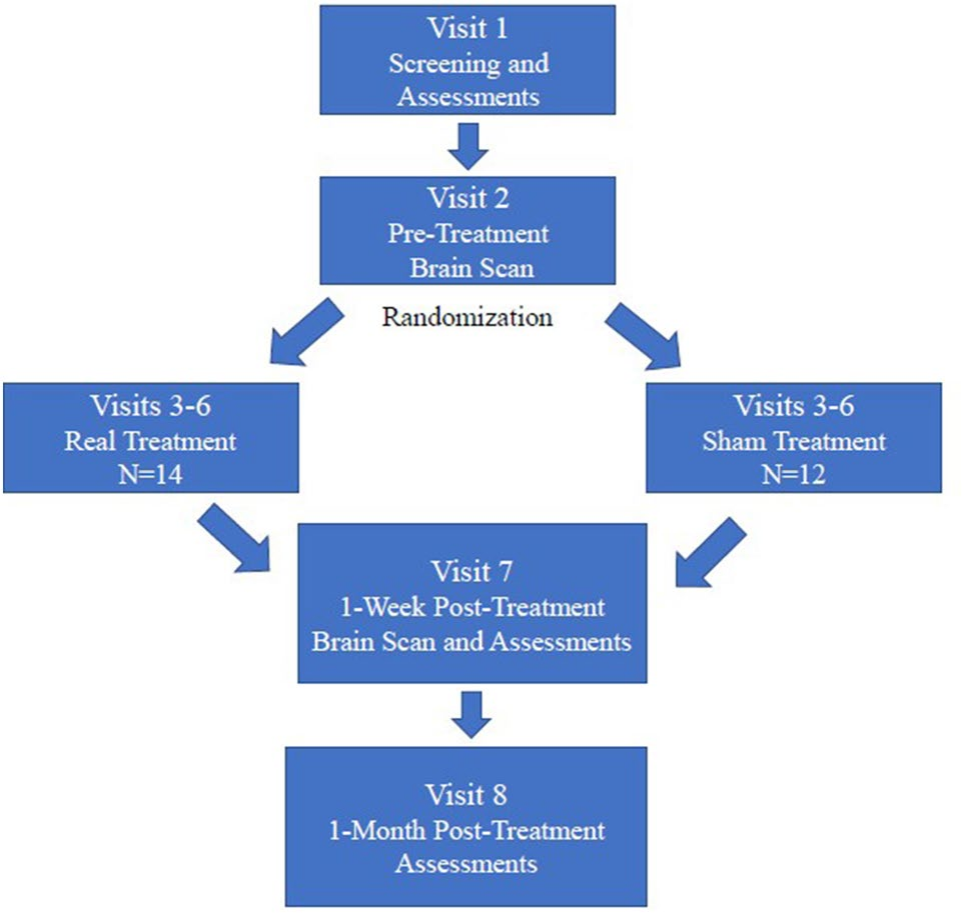
Study protocol flow chart.

The study was conducted according to the following schedule: Visit 1: Screening and assessments; Visit 2: Pre-treatment brain scan; Visit 3–6: Study treatments; Visit 7: Post-treatment 1-week assessments and brain scan; Visit 8: Post treatment 1-month follow up assessments (see Fig. 1).

### MRI design

Brain anatomical scans for use with neuronavigation guided rTMS treatment were obtained with rapid gradient-echo (MP RAGE) sampling (176 slices T1, 256 × 256 and 1 mm slice thickness).

#### Resting state fMRI

Pre and one-week post treatment 5-min resting state fMRI were conducted in a 3 T GE scanner with T2*- weighted EPI-sequence (TE = 30 ms, TR = 2.0 s, a = 90°, TH = 4 mm, 32 slices, FOV = 220 × 220 mm^2^).

##### Evoked heat pain fMRI

Using an identical pulse sequence as the resting state fMRI, the evoked heat pain (HP) paradigm was conducted with a well-established study paradigm^22–24^. An oscillating heat pain stimulus was delivered to the subjects’ left medial calf via a fMRI compatible Peltier thermode (Medoc fMRI Pathway Model ATS, 30 × 30 mm). The stimulus paradigm consists of 20 cycles of 30-s baseline temperature at 32 degrees C alternating with 10-s heat pain stimuli at 46 degrees C.

### Treatment

Following baseline fMRI scanning, the subjects were then treated with four sessions of either active or sham rTMS at > 24 and < 72 h apart according to a computer-generated randomization list with equal probability. Using BrainVoyager (Brain Innovation, Maastricht, Netherlands) TMS neuronavigation software, treatment was delivered to the brain region identified as the left dorsolateral prefrontal cortex, which was marked using coordinates established from a prior functional imaging study^15,25^. For the real group, the MagVenture Pro 30 machine (MagVenture, Inc., Alpharetta, GA, USA) was used to administer rTMS with a figure-of-eight coil. These subjects received 20 trains at 10 Hz with 100 pulses in each train and an inter train interval of 1.0 s, at an intensity of 80% of the resting motor threshold (RMT), a stimulation paradigm in line with previous randomized controlled studies and a recent international expert panel recommendation^15,26^. For the sham group, in order to maintain study blindness, the coil was flipped 180 degrees and wrapped with Giron Magnetic field shield, after which the same rTMS sequence was run. This method of performing sham rTMS has been previously validated and implemented in several of our other studies^15,27,28^.

Within 7 days of the last treatment or sham procedure, a second fMRI scan was conducted in the same fashion as the baseline scan to again assess resting state and HP evoked activity while headache assessments were conducted as well.

### Headache assessments

Two headache characteristics, persistent headache and debilitating headache exacerbation, were assessed during the study. Persistent Headache refers to a chronic daily headache that never goes away. Debilitating Headache refers to the intensity of headache being so severe that it completely debilitates a person's daily normal functions to a level at which he or she may need to resort to bed rest^28,29^. Subjects were provided with a daily headache diary, which they filled out every evening during the study between visits. In the diary, they were asked to report if they had a persistent headache over the last 24 h and rate the average intensity of the headache on a 0–10 Numerical Rating Scale (NRS). In addition, they were asked to report the duration and intensity of any debilitating headache exacerbations, which completely incapacitated their daily functions.

The daily headache intensity was calculated by averaging the NRS scores between visits for persistent headaches. The debilitating headache frequency was determined as the number of episodes per week between visits. For debilitating headache exacerbation, a composite score was generated by multiplying the average duration (hours/episode) of the headache exacerbation by the frequency (episodes per week) and the average intensity (NRS) of the headache exacerbation.

### Data analysis

Our original headache and neuropsychological assessments results were reported in the previously published paper testing hypotheses that real rTMS would significantly decrease MTBI-HA symptoms in comparison to sham treatment^15^. We were able to obtain fMRI data on 26 of these subjects (of the original 29) and the clinical data was reanalyzed using the same data analytical approach to confirm previous findings. First, descriptive statistics and plots were used to assess the normality of the data for the presence of skew and/or outliers, and baseline data from two groups were compared using an Analysis of Variance (ANOVA) for continuous outcomes and Chi-square for categorical data to discern any significant (*P* < 0.01) baseline between-group differences.

Functional imaging data from pre-treatment at Visit 2 and at 1-week post-treatment at Visit 7 were used as the primary outcome measures. For fMRI data analyses, between-group (Real vs Sham and Pre vs. Post) comparisons for both resting and evoked heat pain paradigms were conducted in BrainVoyager QX^30,31^. Preprocessing steps were conducted using a previous established algorithm^25^. Using this software, each brain was anatomically normalized to the Talaraich coordinate plane for homogenous processing. For the resting state fMRI, independent component analysis (ICA) was conducted and spatially guided by the volume of interest (VOI) from the previous study^10^. This analysis used the FastICA algorithm with the deflation approach found in the self-organizing group independent component analysis (SogICA) plug-in provided by BrainVoyager QX^32^. The ICA decomposition was spatially guided based on our region of interest (ROI)/VOI which was established in previous HP-fMRI studies^25,33^. The settings were as follows: group components = 30, absolute threshold before similarity processing = 0, degree of temporal similarity in the clustering = 0. Once organized by the plugin, a two-factor (cluster × within/between treatment group) ANOVA was conducted to determine the interaction. If a significant interaction was detected, a second level analysis and a student’s T-test was then used to discern whether the significant interaction was due to an increase or decrease of activity in the region of interest. In the case of the Evoked HP fMRI, a general linear model (GLM) was first created to model the HP response. A box-car time course modified with the convolution of the two-gamma hemodynamic response function was set as a measurement for the blood oxygen-level dependent (BOLD) signal in the MRI. Similarly, VOIs from the previous study were utilized as areas of interest (see Table 1)^10^. Within-group (Post-REAL > Pre-REAL, Post-SHAM > Pre-SHAM) and Between-Group Random Effect Analyses were conducted. The analytical approach has factored in correction for multiple comparisons in a general linear model^25,33^.

**Table 1:** VOI of previous study in Talairach coordinates.

Region	Mean X	Mean Y	Mean Z	Std. Dev X	Std. Dev Y	Std. Dev Z	Cluster size (Voxels)
R INS	44.05	− 6.81	1.48	6.14	3.61	3.46	993
R INS	36.20	− 15.13	14.11	2.22	8.21	5.61	1753
R DLPFC	24.01	22.45	7.99	2.76	5.29	2.65	498
R SSC2	8.40	− 54.54	57.95	4.44	5.31	3.06	944
R THLMS	5.10	− 13.99	3.07	7.84	6.60	5.61	3462
R ACC	− 4.08	− 5.08	39.15	6.13	15.07	3.86	4797
R PONS	0.76	− 25.31	− 20.39	2.75	5.32	4.23	1076
L BA11	− 30.80	8.05	− 0.19	11.25	7.89	10.25	11,841
L THLMS	− 23.76	− 21.65	− 3.58	7.94	2.66	3.84	1366
L PFC	− 32.95	31.70	30.25	3.55	5.31	3.87	871

Several regions that were seen in a previous study linked with pain modulation with specific Talairach coordinates. Indication of left or right given as L or R respectively.

*INS* insula, *DLPFC* dorsal lateral prefrontal cortex, *SSC* somatosensory cortex, *THLMS* thalamus, *ACC* anterior cingulate cortex, *PFC* prefrontal cortex, *BA* Brodmann Area.

Headache data from baseline assessments at Visit 1, post-treatment 1-week assessments at Visit 7, and post treatment 1-month follow up assessments at Visit 8 were compared across visits using a 2-factor (treatment × visit) Repeated Measures Analysis of Variance (RM-ANOVA). To further test for any lingering treatment effect as noted in the previous study of this cohort, pairwise tests were conducted to compare 1-week and 4-week results with baseline, with Bonferroni correction. For headache outcomes, all data analyses were performed using SPSS version 23 software.

### Ethics approval and consent to participate

All research subjects provided informed consent for the study. The research protocol was approved by the Veteran’s Affairs San Diego Healthcare System Institutional Review Board for Human Research Protection.

## Results

### Demographic

From May 2014 to June 2016, a total of 29 subjects were screened and enrolled in the study and 26 subjects, 6 females and 20 males, completed their participation by the end of the funding period, with 3 subjects withdrawn due to loss of post-treatment imaging. Of the 14 subjects who completed the study in the “Real” group, 2 were female and 12 were male, average age (years ± SD) was 33 ± 8 years, and average duration (months ± SD) of MTBI-related headache was 95 ± 83. Of the 12 subjects who completed the study in the “Sham” group, 4 were female and 8 were male, average age (years ± SD) was 35 ± 8, and average duration (months ± SD) of MTBI-related headache was 99 ± 58. Overall, there are no significant demographic differences between the two groups. In addition, baseline medication use and resting motor thresholds between the two groups showed no significance (see Table 2). The previous publication reported full analyses of the clinical data of the 29 subjects^15^. However, as fMRI data was not available on 3 of the subjects, we replicated the previously reported clinical headache data analysis to confirm reported results. These analyses are not part of hypothesis testing for this current report.

**Table 2:** Demographic information.

	Real (N = 14)	Sham (N = 12)	Real vs Sham *P*-value
**Gender**			
Males	12	8	–
Females	2	4	–
**Cause of TBI**			
Blast	5	5	–
Non-blast	7	5	–
Both	2	2	–
**Duration in months of MTBI-HA ± SD**	95 ± 83	105 ± 61	–
**Number of subjects using medications**			
Triptans	5	7	–
TCA	8	6	–
NSAID	3	5	–
Gabapentanoid	4	1	–
Acetaminophen	1	1	–
SSRI	0	2	–
AED	0	3	–
Opioid	0	1	–
**Average age ± SD**	33.0 ± 8	35.6 ± 8	–
**Average RMT ± SD**	55.5 ± 13.7	52.6 ± 8.7	–

*RMT* Resting Motor Threshold;—no significant difference between groups.

### Functional imaging results

FMRI analysis showed increased resting state and evoked heat pain activities at the LPFC in the real group after active rTMS treatment. In resting state, the sham group showed a decrease in activity after sham rTMS treatment.

#### Resting state

SogICA was conducted to assess whether at resting state, LPFC as a selected region of interest (ROI) would demonstrate a significant interaction in treatment and whether the significant interaction was due to a preexisting baseline between-group difference or a change of activity in the ROI. A cut-off value of *P* < 0.01 was chosen for significance in this study, thus incorporating type I correction for multiple variable analysis. A minimum cluster size threshold of 100 voxels was chosen^10^. Between-Group Random Effect Analyses, which internally adjusted for multiple comparisons, were used for the resting fMRI data analyses.

### Pre-treatment between-group resting state comparison

In the selected ROI/VOI, there was no significant between-group interaction in resting state between the REAL and SHAM groups at baseline.

### Post-treatment within- and between-group resting state comparison

#### Post-real versus pre-real within-group

**Figure 2:**
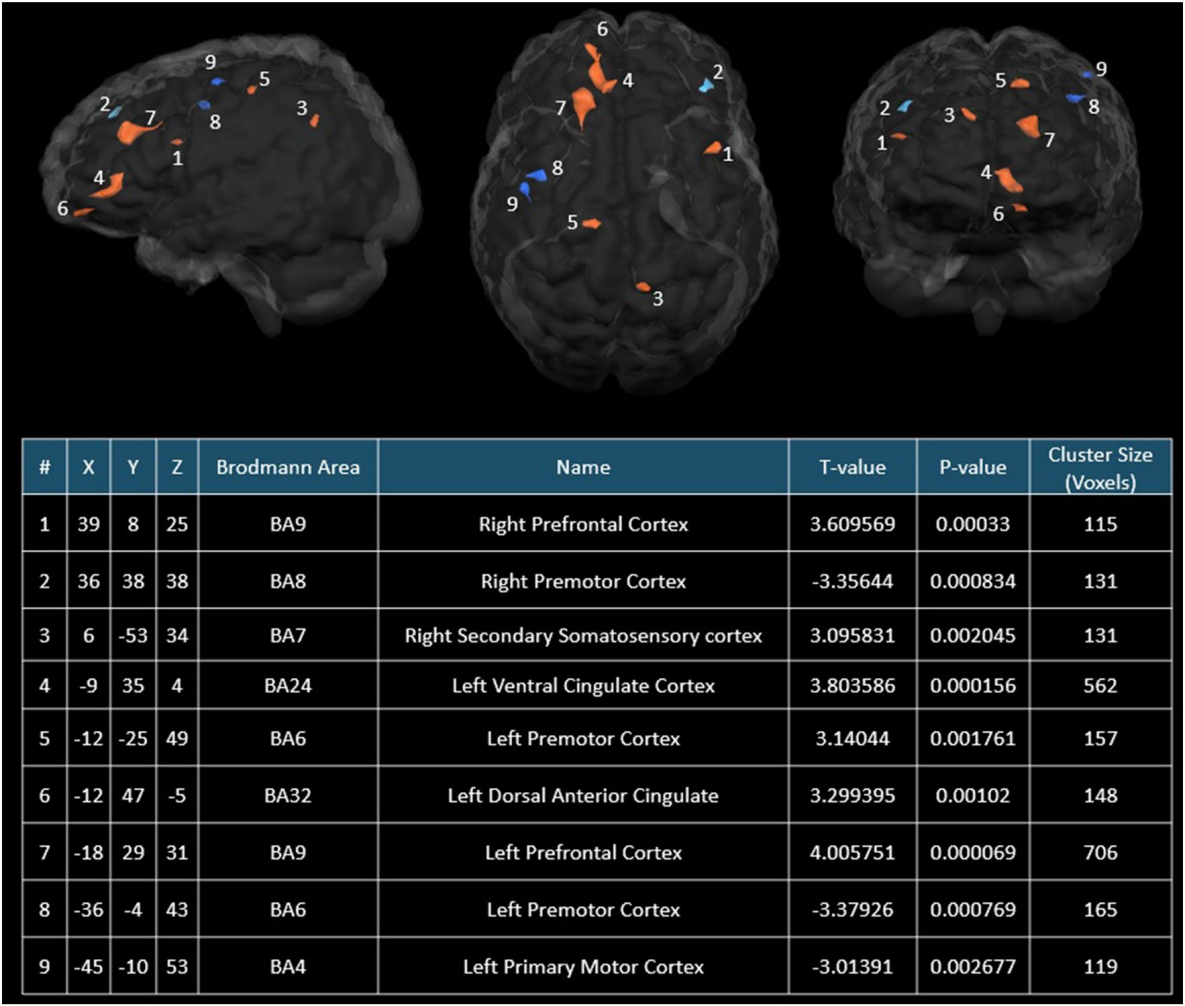
Real treatment related resting state functional connectivity differences with LPFC as the seeded modulatory function region. Regions in brown–red indicated significant (*P* < 0.01; Cluster size > 100 voxels) more functional connectivities to the LPFC in the Post-REAL group in comparison to Pre-REAL group, whereas regions in blue represented significant (*P* < 0.01; Cluster size > 100 voxels) less functional connectivities to the LPFC in the Post-REAL group in comparison to Pre-REAL group. Glass brains were created using BrainVoyager QX 2.8.

In the REAL group, a T-test demonstrated a significant (T-value = 4.01, *P* < 0.0001) increase in connectivity in the LPFC after the treatment (see Fig. 2).

#### Post-SHAM versus pre-SHAM within-group

**Figure 3:**
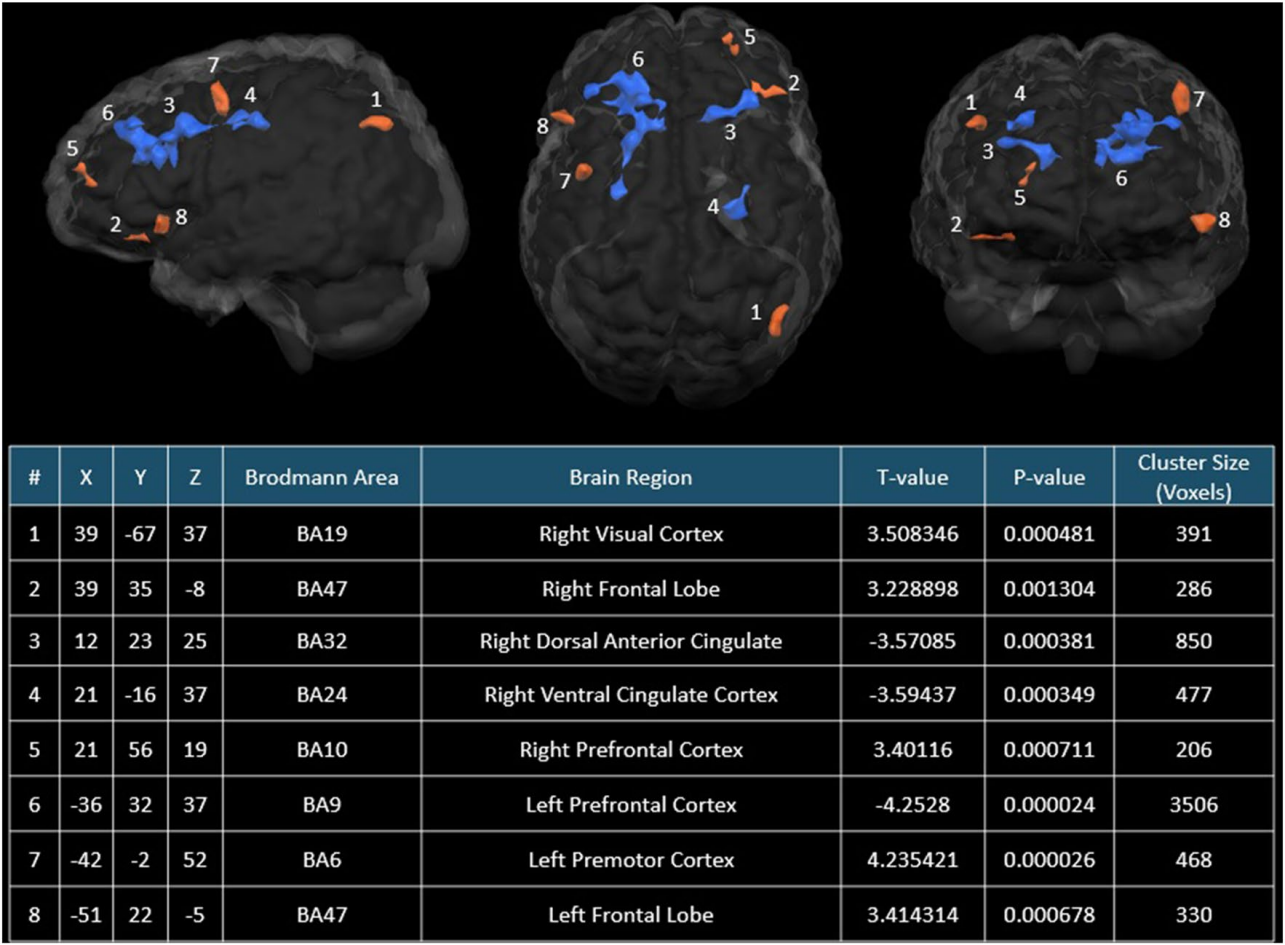
Sham treatment related resting state functional connectivity differences with LPFC as the seeded modulatory function region. Regions in brown–red indicated significant (*P* < 0.01; Cluster size > 100 voxels) more functional connectivities to the LPFC in the Post-SHAM group in comparison to Pre-SHAM group, whereas regions in blue represented significant (*P* < 0.01; Cluster size > 100 voxels) less functional connectivities to the LPFC in the Post-SHAM group in comparison to Pre-SHAM group. Glass brains were created using BrainVoyager QX 2.8.

In the SHAM group, the resting state functional connectivity analyses also revealed a significant (F = 3.89, *P* < 0.0001) interaction between the LPFC and the visits. The T-test demonstrated a significant (T-value = − 4.25, *P* < 0.0001) decrease in activity in the LPFC following sham intervention (see Fig. 3).

#### Post-REAL versus post-SHAM between-group

In the LPFC, there was no significant difference in resting state connectivity between the REAL and SHAM groups.

#### Evoked heat pain

Between-Group Random Effect Analyses, which internally adjusted for multiple comparisons, were used for the evoked HP fMRI data analyses.

##### Pre-treatment between-group evoked HP comparison

For the LPFC, no significant difference was found between the REAL and SHAM groups at baseline.

##### Post-treatment Within- and Between-Group Evoked HP Comparisons

##### Pre-REAL vs. post-REAL within-group

For the LPFC, following real rTMS, a significant (T-value = 3.19, *P* < 0.002, see Table 3) increase in activity was observed in the same ROI.

**Table 3:** Left prefrontal cortex evoked HP fMRI comparisons.

	F value	*P* value	T value	*P* value
Pre-REAL – Pre-SHAM	0.03	0.872	− 0.25	0.800
Post-REAL – Pre-REAL	5.66	0.025	3.19	**< 0.002**
Post-SHAM –Pre-SHAM	0.43	0.520	− 1.08	0.281
Post-REAL – Post-SHAM	2.92	0.102	1.66	0.100

##### Pre-SHAM vs. Post-SHAM within-group

The SHAM group underwent no statistically significant (T = − 1.08, *P* = 0.281, see Table 3) change in activity in the LPFC during the evoked HP fMRI scan.

##### Post-SHAM vs. post-REAL between-group

In comparing post-real versus post-sham LPFC activity during the evoked HP fMRI scan, no significant difference was found.

##### Clinical data analysis results

Replicated analysis of the headache clinical data in the subset of subjects who completed pre- and post- fMRI scans confirmed previous findings on improvement of headaches for debilitating headache intensity, daily headache intensity, and the prevalence of persistent headaches for subjects in the real group following treatment^15^.

### Headache assessments

#### Debilitating headache

**Figure 4:**
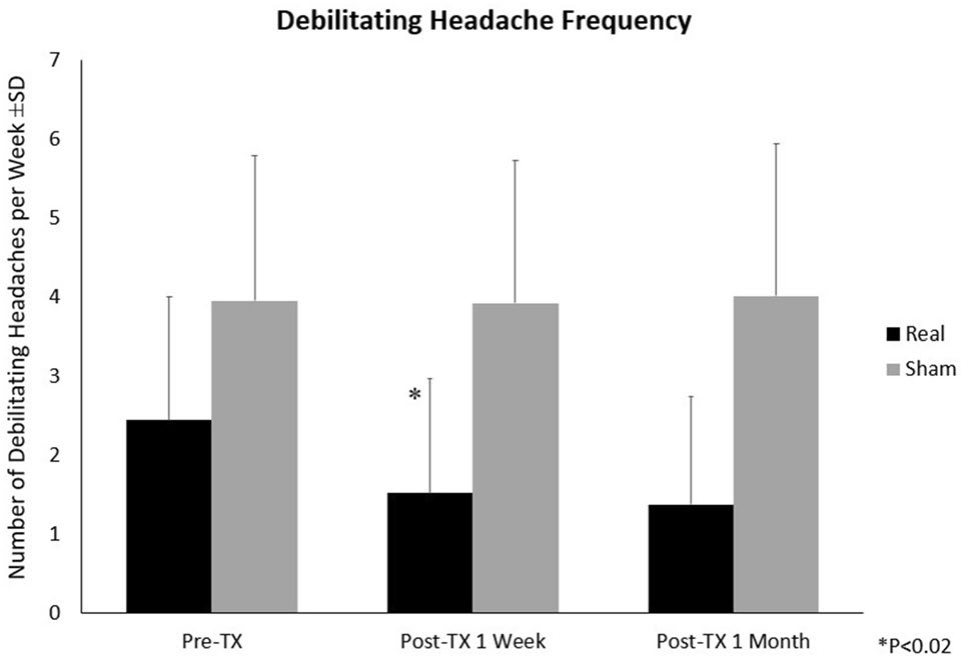
Debilitating Headache Frequency. Pre-Tx: Pre-treatment; Post-Tx: Post-treatment; *P < 0.02. Analysis was conducted using SPSS version 23.

A significant interaction was found between treatment and visit (f_2,48_ = 3.18, *p* = 0.050) and there was a significant difference between treatment groups when baseline was compared with 1 week (f_1,24_ = 7.11, *p* = 0.014) for debilitating headache frequency. The average debilitating headache frequency (number of debilitating headache episodes per week ± SD) at one-week post-treatment was significantly lower in the REAL group (1.52 ± 1.45 from baseline 2.44 ± 1.56) than the SHAM group (3.92 ± 1.80 from baseline 3.95 ± 1.83). There was a strong trend towards a difference between treatment groups from baseline to 4 weeks (f_1,24_ = 3.99, *p* = 0.057) (see Fig. 4, Table 4). For the debilitating headache composite scores, no significance was found in this subset of subjects.

**Table 4:** Table of means (± SD) of headache assessments and significant findings.

	Real ± SD	Sham ± SD		df	F	*p*-value
**Debilitating headache frequency**						
Baseline	2.44 ± 1.56	3.95 ± 1.83	Overall Treatment versus Visit	2,48	3.18	**0.050**
1-Week Post-TX	1.52 ± 1.45	3.92 ± 1.80	Baseline versus 1-Week Post-TX	1,24	7.11	**0.014**
4-Week Post-TX	1.38 ± 1.37	4.01 ± 1.92	Baseline versus 4-Week Post-TX	1,24	3.99	0.057
**Daily headache intensity**						
Baseline	4.9 ± 1.7	4.9 ± 1.4	Overall Treatment versus Visit	2,48	9.24	** < 0.001**
1-Week Post-TX	3.5 ± 2.0	4.8 ± 1.4	Baseline versus 1-Week Post-TX	1,24	17.29	** < 0.001**
4-Week Post-TX	3.6 ± 2.0	4.8 ± 1.6	Baseline versus 4-Week Post-TX	1,24	9.91	**0.004**
**Prevalence of persistent headaches**						
Baseline	1.00 ± 0.00	1.00 ± 0.00	Overall Treatment versus Visit	2,48	3.50	**0.038**
1-Week Post-TX	0.50 ± 0.52	0.92 ± 0.29	Baseline versus 1-Week Post-TX	1,24	6.10	**0.021**
4-Week Post-TX	0.43 ± 0.51	0.75 ± 0.45	Baseline versus 4-Week Post-TX	1,24	2.82	0.106

Post-TX: post-treatment.

The bold values represent significance of *p* < 0.05

#### Daily headache intensity

**Figure 5:**
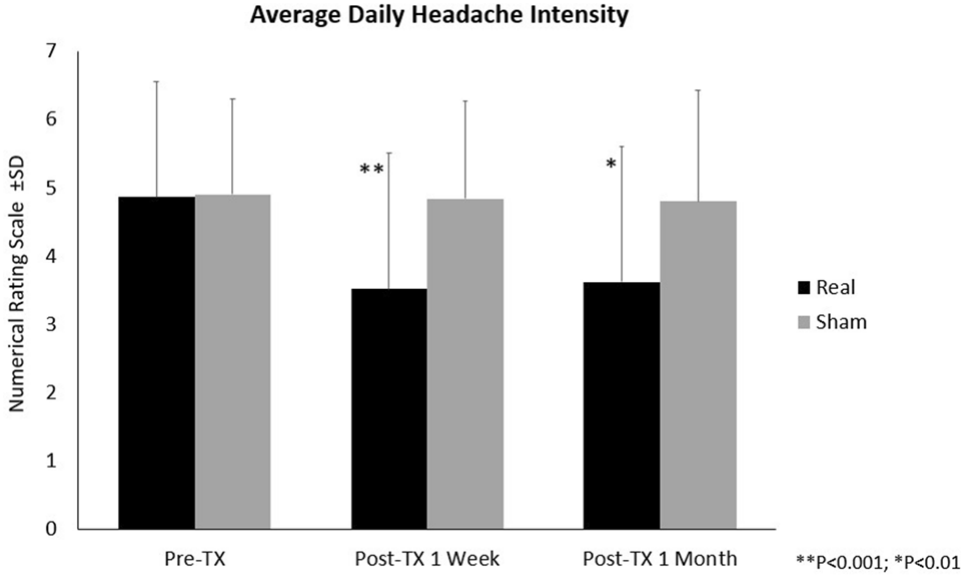
Average Daily Headache Intensity. Pre-Tx: Pre-treatment; Post-Tx: Post-treatment; **P < 0.001; *P < 0.01. Analysis was conducted using SPSS version 23.

A two-factor (treatment × visit) RM-ANOVA indicated an overall significant (f_2,48_ = 9.24, *p* < 0.001) interaction for the average daily persistent headache intensity. In the pre- and post-treatment one week comparison, the REAL group exhibited a significant (f_1,24_ = 17.29, *p* < 0.001) 25% reduction in the average daily headache intensity score ± SD (from 4.9 ± 1.7 to 3.5 ± 2.0), whereas the SHAM group had no significant change in daily headache scores (from 4.9 ± 1.4 to 4.8 ± 1.4). From baseline to four-week post-treatment, the REAL group experienced a significant 23% decrease (f_1,24_ = 9.91, *p* = 0.004) in the average headache intensity scores (from 4.9 ± 1.7 to 3.6 ± 2.0) while the SHAM group showed no significant change in the scores (from 4.9 ± 1.4 to 4.8 ± 1.6) (see Fig. 5, Table 4).

#### Prevalence of persistent headaches

**Figure 6:**
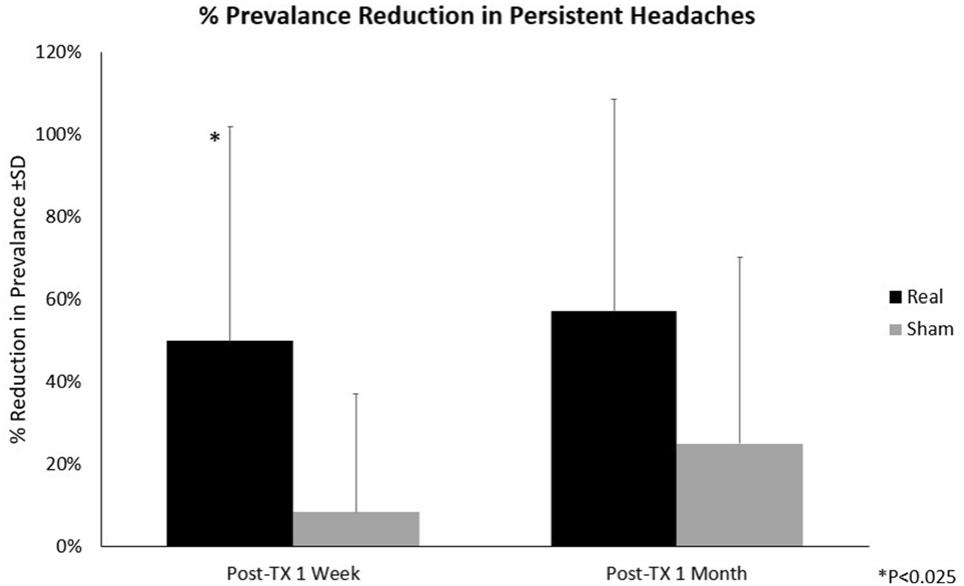
% Reduction in the Prevalence of Persistent Headaches. Pre-Tx: Pre-treatment; Post-Tx: Post-treatment; *P < 0.025. Analysis was conducted using SPSS version 23.

A two-factor (treatment × visit) RM-ANOVA indicated an overall significant interaction (f_2,48_ = 3.50, *p* = 0.038) for the prevalence of persistent headaches. While the pre-treatment prevalence of persistent headaches was the same in both study groups (100%), a significant (f_1,24_ = 6.10, *p* = 0.021) interaction was found when comparing groups at the one-week post treatment visit versus baseline. 50% of subjects in the REAL group no longer experienced persistent headaches at the one-week post-treatment assessment in comparison to 8% of the SHAM group. However, there was no significant interaction (f_1,24_ = 2.82, *p* = 0.106) found at the four-week post-treatment assessment (though there was a 57% prevalence reduction in the REAL group versus 25% reduction in the SHAM group) (see Fig. 6, Table 4).

#### Neuropsychological assessments/side effects

Aside from the previously reported transient significant increase in the Perseverations component of the Connors CPT-II attention assessment in the REAL group, no other significant neuropsychological changes or side effects were reported by the study cohort^15^.

## Discussion

The human cerebral cortex is increasingly being appreciated as an important component in the pain modulation system^34^. Specifically, it has been shown that “top-down” modulation of the ascending midbrain-thalamic-cingulate pain pathway may depend heavily on activity originating in the prefrontal cortex^35^. The left dorsolateral prefrontal cortex (DLPFC), in particular, has been shown to exert important modulatory activity onto the insula and thalamus, and decreases in such connectivity have been associated with uncontrollable and chronic pain. Diminished DLPFC gray matter has also been associated with several chronic pain conditions^36^. Even interhemispheric connectivity between left and right DLPFC has been associated with altered pain tolerance and sensitivity^37^. Dysfunction of these supraspinal pain modulatory functional connectivities, particularly involving the PFC, is a contributing factor in a variety of central neuropathic pain states^38^, and it has been demonstrated that headache associated with mild traumatic brain injury may be an example of this phenomenon^10,39^. The current study begins to elucidate, functionally, how rTMS might be affecting both resting and evoked pain state activity of the LPFC, a brain area known to have this pain modulatory function, and therefore one possible mechanism for how it may produce its apparent clinical benefit in MTBI-HA.

Cortically applied rTMS has been shown to exert a variety of effects on pain signaling and modulation. Compared to sham treatment, rTMS increases mechanical and thermal pain thresholds, alters signaling levels in several important pain pathway areas, including the posterior cingulate gyrus, insula, anterior cingulate cortex, thalamus, midbrain, and medulla, and has positive impacts on a variety of pain syndromes and states, including fibromyalgia, migraine, neuropathic pain, and even acute post-surgical visceral pain, among others. The antinociceptive mechanisms of rTMS are thought to involve opioid and NMDA signaling, as indicated by inhibition of these effects by administration of exogenous opioids and ketamine, respectively, with the latter also suggesting the involvement of long-term potentiation^34^. Moreover, depending on the cortical target, rTMS has the ability to not only impact the rated intensity of pain, but also the perceived control over pain, emotional component of the pain experience, the unpleasantness of pain, the adaptation to pain, pain tolerance, the localization of pain, and pain resolution, underscoring the incredibly complex relationships between the many supraspinal pain modulation areas as elucidated by rTMS-induced experimental virtual lesioning^40^.

In the current study, the group of subjects undergoing real rTMS to the DLPFC demonstrated a significant improvement in resting state activity in the LPFC. Conversely, the same cortical region showed significant negative change in activity following sham intervention. The findings offer several insights into the potential mechanisms of rTMS in the treatment of MTBI-associated headache. The observed increase in resting state activity in pain modulatory regions following rTMS is promising and consistent with observed clinical outcomes of decreased headache prevalence and intensity in both current and previous data analyses^15^. The finding of decreased resting state activity at the LPFC following sham rTMS is surprising, especially given the valid concern for likely positive placebo effects of sham rTMS in general^41^. Possible explanations for this may include untreated disease progression in the sham group, unintended sensory side effects of sham rTMS, or even, theoretically, a nocebo effect in this group. More studies are required to determine the likelihood and extent of these impacts in this patient population and with sham rTMS in general^41^.

Perhaps the most notable result is the statistically significant increase in activity in the LPFC during evoked heat pain fMRI scan following real rTMS therapy, whereas the same was not true in the sham group (Table 3). This suggests an increased activation of the known pain modulation pathway mediated by the left prefrontal cortex following rTMS and provides a possible explanation for the observed clinical benefit of the therapy.

One notable limitation of the study is that, while the evoked heat pain method utilized in the study operates via the standard somatic pain signaling pathway, headache in general, and particularly MTBI-HA, is mediated by less well understood but clearly different mechanisms. The mechanisms behind headache in general depend on the type of headache and are still being extensively studied, but include a variety of neural, meningeal, muscular, and vascular etiologies. MTBI-HA is even less well understood but, as described previously, is likely a neuropathic pain state induced via a combination of diffuse axonal injury and its effects on supraspinal pain modulation. While the current study represents a step toward better understanding this pain pathway, the authors recognize that the evoked heat pain method used here activates a very likely related but still different one.

While significant treatment-related within-group changes in resting state activities and evoked heat pain responses from the PFC were noted, no statistically significant between-group post-treatment differences were noted. This is most likely due to the small sample size of the study. Thus, future studies with larger sample sizes should be conducted to further investigate the mechanisms underlying the therapeutic effect of rTMS in MTBI-HA patients.

Though the current study did not detect any significant and sustained changes in neuropsychological assessments, it did show headache symptomology improvement, supporting the notion that the observed treatment-related supraspinal functional connectivity improvement may be directly related to the observed sustained analgesic benefits of the treatment. However, future studies with larger sample sizes should be conducted to further assess the relationship between the neuroimaging findings and headache symptoms.
